# Cooperation of Notch and Ras/MAPK signaling pathways in human breast carcinogenesis

**DOI:** 10.1186/1476-4598-8-128

**Published:** 2009-12-23

**Authors:** Suruchi Mittal, Deepa Subramanyam, Devaveena Dey, Rekha V Kumar, Annapoorni Rangarajan

**Affiliations:** 1 Department of Molecular Reproduction, Development and Genetics, Indian Institute of Science, Bangalore 560 012, Karnataka, India; 2 National Centre for Biological Sciences, Bangalore, Karnataka, India; 3 Department of Urology, University of California at San Francisco, San Francisco, CA-94143, USA; 4 Department of Pathology, Kidwai Memorial Institute of Oncology Bangalore 560 029, India

## Abstract

**Background:**

Recent studies have implicated aberrant Notch signaling in breast cancers. Yet, relatively little is known about the pattern of expression of various components of the Notch pathway, or its mechanism of action. To better understand the role of the Notch pathway in breast cancer, we have undertaken a detailed expression analysis of various Notch receptors, their ligands, and downstream targets at different stages of breast cancer progression.

**Results:**

We report here that there is a general increase in the expression levels of Notch 1, 2, 4, Jagged1, Jagged2, and Delta-like 4 proteins in breast cancers, with simultaneous upregulation of multiple Notch receptors and ligands in a given cancer tissue. While Notch3 and Delta-like1 were undetectable in normal tissues, moderate to high expression was detected in several cancers. We detected the presence of active, cleaved Notch1, along with downstream targets of the Notch pathway, Hes1/Hes5, in ~75% of breast cancers, clearly indicating that in a large proportion of breast cancers Notch signaling is aberrantly activated. Furthermore, we detected cleaved Notch1 and Hes1/5 in early precursors of breast cancers - hyperplasia and ductal carcinoma *in situ *- suggesting that aberrant Notch activation may be an early event in breast cancer progression. Mechanistically, while constitutively active Notch1 alone failed to transform immortalized breast cells, it synergized with the Ras/MAPK pathway to mediate transformation. This cooperation is reflected *in vivo*, as a subset of cleaved Notch positive tumors additionally expressed phopsho-Erk1/2 in the nuclei. Such cases exhibited high node positivity, suggesting that Notch-Ras cooperation may lead to poor prognosis.

**Conclusions:**

High level expression of Notch receptors and ligands, and its increased activation in several breast cancers and early precursors, places Notch signaling as a key player in breast cancer pathogenesis. Its cooperation with the Ras/MAPK pathway in transformation offers combined inhibition of the two pathways as a new modality for breast cancer treatment.

## Background

Breast cancer is a leading cause of cancer-related death in women the world over, particularly in the Western population. However, emerging trends indicate an alarming rise in breast cancer incidences in other parts of the world too [1]. Investigations into the molecular mechanisms and signaling pathways leading to breast cancer pathogenesis have over the years resulted in the discovery of drugs to treat subsets of breast cancers. Yet, the significant number of breast cancer-associated deaths each year warrants further investigations into the mechanisms of disease progression and identification of key players involved therein, ultimately leading to better treatment strategies.

A number of signaling pathways, including Her-2, EGFR and Wnt, have been implicated in the progression of breast cancer [2,3], with the Notch pathway being associated with this process more recently [4]. Notch proteins are cell-surface receptors activated by interaction with cell-surface ligands of the Jagged/Delta family. The mammalian family of Notch receptors consists of four members: Notch1 through Notch4, while the ligand family consists of five members: Jagged 1, 2, Delta like 1 (Dll1), Delta-like 3 (Dll3) and Delta-like 4 (Dll4) [4,5]. In the absence of ligand binding, Notch receptors are inactive. However, binding of the ligand to the Notch receptor induces site-specific cleavage resulting in the release of the Notch Intracellular Domain (NICD). This NICD, or cleaved Notch protein, translocates to the nucleus where it modulates gene expression through interaction with members of the CSL (CBF-1, Suppressor of Hairless, Lag-1) family of transcription factors. Notch activation leads to elevated expression of specific genes including Hes1 and Hes5 [6]. However, CSL-independent, deltex-dependent, cytosolic functions of Notch have also been reported [7]. Notch signaling is involved in regulating a wide range of cellular activities involving cell differentiation, proliferation, survival, and more recently, in the maintenance of stemness [8-10].

The association of Notch signaling with human carcinogenesis has been well documented in the literature [11]. First identified in a small subset of human T-cell acute lymphoblastic leukemias as a chromosomal breakpoint [12], activating Notch mutations have now been detected in almost 50% of these cancers [13], suggesting a strong causal relationship between Notch activation and disease development. However genetic lesions of the *Notch *loci have so far not been detected in solid cancers [14]. Yet, aberrant expression, and activation of the pathway, have been reported in several human cancers including multiple myeloma, pancreatic cancer, cancers of prostate, cervix, colon, lung, skin, and brain [15-21].

More recently Notch signaling has been implicated in human breast cancers. Co-expression of high levels of Jagged1 and Notch1 was associated with poor survival in breast cancers [22]. Accumulation of the intracellular domain of Notch1 has been detected in a variety of breast cancers [23]. Interestingly, despite the expression of Numb, a negative modulator of Notch activity, the Notch pathway was found to be active in several breast cancers [24]. Functionally, over-expression of constitutively active Notch led to the *in vitro *transformation of MCF-10A, an immortalized breast epithelial cell line [23]. Functional synergy between Notch and Myc was also shown to lead to the development of mammary cancers [25], indicating that Notch signaling may cooperate with other known oncogenes in promoting mammary carcinogenesis.

Furthermore, inhibition of Notch signaling has been shown to hinder the survival of breast cancer stem cells [26,27], as well as reverse the tumorigenic potential of breast cancer cell lines [23]. Taken together, these data suggest that Notch signaling may play a key role in breast cancer pathogenesis. Yet, a clear understanding of the specific Notch receptors and ligands involved in this process, and the mechanisms of Notch action, are still lacking.

In order to better understand the role of the Notch pathway in breast carcinogenesis, we have undertaken a detailed immunohistochemical analysis to determine the expression of the Notch family members during different stages of breast carcinogenesis. Our results indicate that there is a general increase in the expression of several Notch pathway members in breast cancers compared to normal breast tissue. Additionally, our results reveal a strong correlation between the expression of cleaved Notch1 and downstream targets in ~75% of cancers analyzed, indicating an aberrant activation of the Notch pathway in a vast majority of breast cancers. Moreover, this co-expression was observed as early as hyperplasia and ductal carcinoma *in situ*, suggesting that aberrant activation of the Notch pathway may be an early event in breast cancer progression. Furthermore, *in vitro *transformation assays, as well as immunohistochemistry in naturally occurring breast cancers, revealed a co-operation between Notch and Ras/MAPK pathways.

## Results

### Elevated expression of Notch receptors and ligands in breast cancer

In order to understand the role of Notch signaling in breast carcinogenesis, we sought to identify which Notch receptors and ligands were expressed in normal and cancerous breast tissues. We first carried out an RT-PCR analysis for the transcripts of Notch1, Notch2, Notch3, Notch4, Jagged1, Jagged2, Delta-like1(Dll1), Delta-like3 (Dll3), and Delta-like4 (Dll4) from several immortalized and transformed breast cell lines (Additional file 1: Table 1) We were able to detect transcripts of all members of the Notch family, except for Dll3 in diverse immortalized and cancer cell lines. To better understand the spatial expression pattern of the proteins encoded by these transcripts within intact breast tissue, we undertook a detailed immunohistochemical analysis of normal and cancerous tissue from patient biopsies. All cancers analyzed were grade 3 invasive ductal carcinomas. We graded the expression levels of each of the antigens using an arbitrary scale of 1+ to 4+ (where 1+ is lowest and 4+ is highest staining) as shown in Additional file 2: Fig. 1.

**Figure 1:**
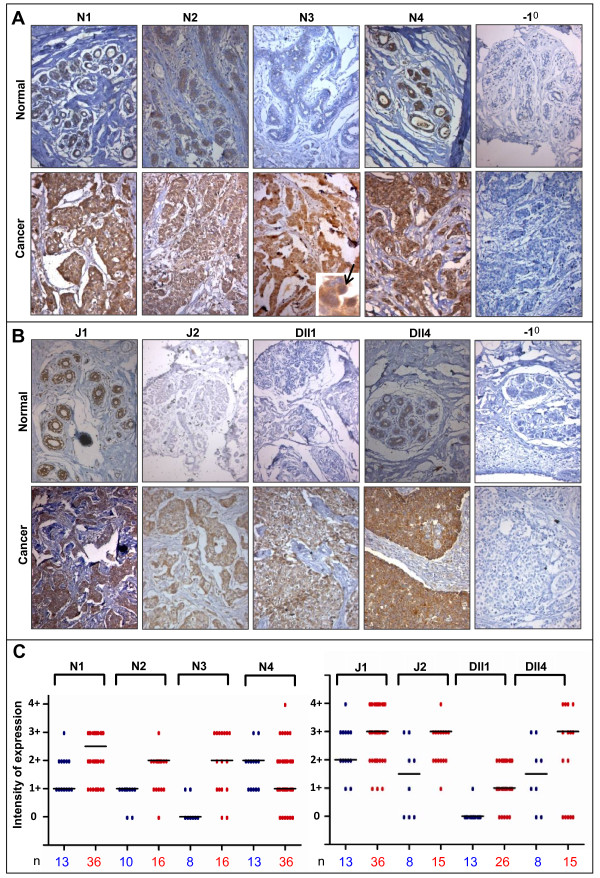
**Overexpression of Notch receptors and ligands in breast cancer**. Photomicrographs represent staining of normal and cancerous breast tissue sections with antibodies that recognize **(A) **Notch1, Notch2, Notch3, Notch4, and **(B) **Jagged1, Jagged2, Delta-like 1 and Delta-like 4. Inset and arrow in **(A) **shows nuclear localization of Notch3 in breast cancers. Sections stained in the absence of primary antibodies served as negative controls (-1^0^). Samples were counterstained with haemotoxylin, and images taken at a magnification of 20×. **(C) **Scatter plot represents expression of Notch receptors and ligands across various normal (blue) and cancer (red) breast tissue samples analyzed. The total number of cases analyzed under each category (n) is mentioned below each column, and the black bar represents the median score.

We detected Notch1 expression in both normal and cancerous breast tissue, with cancer samples showing a higher level of expression. While Notch1 showed a predominantly low expression (1+ or 2+) in normal tissues, in cancers it was expressed at 3+ levels (Fig. 1A and 1C). Similarly, compared to normal tissue, there was a slight increase in the expression levels of Notch2 in cancers. There was no significant change in the expression of Notch4 between normal and cancer tissues; however, the levels of Notch4 was the highest amongst all Notch receptors in normal tissue. Interestingly, while we failed to detect expression of Notch3 proteins in normal tissue, ~88% of breast cancers (14/16) showed Notch3 expression at varying intensities from 1+ to 3+ (Fig. 1A and 1C). This result corroborated with our RT-PCR analysis, where we failed to see transcripts of Notch3 in the immortalized cell lines MCF-10A, HBL-100, and HMLE, whereas, the cancer cell lines MDA-MB 453, T47D and HMLER had detectable levels of Notch3 transcripts (Additional file 1). Surprisingly, we also detected intense nuclear staining for Notch3 in some breast cancers using the N-terminal specific antibody (inset in Fig. 1A), suggesting possible nuclear translocation of full length Notch3. Similar nuclear localization has been previously observed for Notch4 [28] and epidermal growth factor receptor (EGFR) in cancers [29]; however, their significance remains unknown currently.

Upon analysis of the expression pattern of Notch ligands, we found Jagged1 expression varying between intensities of 1+ and 4+ in both normal and cancer tissues (Fig. 1B). However, while 46% of normal tissues analyzed showed high expression levels of Jagged1 (staining intensities of 3+ and 4+), ~70% of cancers showed this level of staining (Fig. 1C), indicating that a greater number of breast cancer samples express Jagged1 at high levels. Jagged2 expression was also found to be higher in cancers with 53% cases having staining intensities of 3+ and 4+ compared to only 25% of normal cases showing this level of staining (Fig. 1B and 1C). Interestingly, while we failed to detect any significant expression of Dll1 in normal breast tissue (with the exception of one tissue which showed low level expression ) ~81% of breast cancers showed a 1+ or 2+ staining for Dll1 (Fig. 1B and 1C). Since we failed to detect Dll3 by RT-PCR in any of the breast cell lines analyzed (Additional file 1: Table 1), immunohistochemistry was not performed for this antigen. Similar to Jagged1 and 2, Dll4 expression was also higher in cancer, with ~53% of cancer cases displaying higher intensities of staining (3+ and 4+) for Dll4, while only 25% of normal cases displayed such high intensity staining (Fig. 1B and 1C). Taken together, these results reveal that there is an overall increase in the expression of several Notch receptors and ligands in breast cancers compared to normal breast tissue, and multiple Notch receptors and ligands are upregulated simultaneously in a given cancer tissue (Additional file 3: Fig. 2).

### Functional activation of the Notch pathway in breast cancers

**Figure 2:**
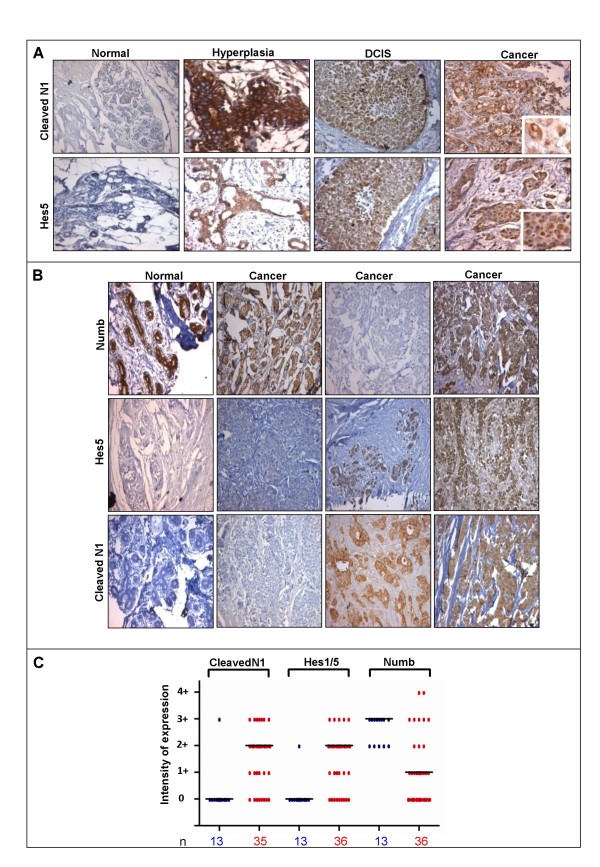
**Activation of the Notch pathway during breast cancer progression**. **(A) **Photomicrographs represent staining of normal, hyperplastic, DCIS, and breast cancer tissue sections with antibodies that specifically recognize the cleaved and active form of Notch1, and Hes5; magnification 20×. Insets show nuclear staining at higher magnification (40×). (**B**) Photomicrographs represent staining of normal and cancerous breast tissue with antibodies against Numb, Hes5 and Cleaved Notch1; magnification 20×. (**C**) Scatter plot represents expression of cleaved Notch1, Hes1/5, and Numb across various normal (blue) and cancer (red) breast tissues analyzed. The total number of cases analyzed under each category (n) is mentioned below each column, and the black bar represents the median score.

Since mere expression of the receptors and ligands does not imply activation of the pathway, we undertook experiments to determine the activation status of the Notch pathway. To do so, we assessed the presence of cleaved, intracellular Notch1 (NICD), produced only after activation of the pathway through interaction of Notch receptors with ligands, using an antibody that specifically detects this form of the protein. Since Notch activation leads to the transcriptional up-regulation of downstream targets such as the Hes family of transcriptional repressors [30], we additionally analyzed the expression of Hes1 and/or Hes5. As many as 75% of breast cancers showed accumulation of cleaved Notch1 (27/35) and expression of Hes1/5 (27/36) (Fig. 2A and 2C). However, with the exception of only one tissue, we failed to detect any of these proteins in normal tissues (Fig. 2A). Indeed, normal ducts adjacent to cancerous areas were negative for cleaved Notch1 and Hes5 (Additional file 4: Fig. 3). For both cleaved Notch1 and Hes1/5 we found nuclear as well as cytoplasmic staining, in accordance with previous publications using the same antibody [31]. Thus, these results clearly indicate that the Notch pathway is functionally active in a large proportion of breast cancers.

In order to know at what stage of cancer progression the Notch pathway gets activated, we analyzed cases of hyperplasia and ductal carcinoma *in situ *(DCIS) which are precursors to invasive ductal carcinomas. We detected nuclear staining for cleaved Notch1 and Hes5 in DCIS tissues (Fig. 2A). Interestingly, a specimen of breast epithelial hyperplasia also showed cleaved Notch1 and Hes5 positivity (Fig. 2A), indicating that Notch signaling might be activated at a very early stage of breast cancer progression.

### Analysis of Numb expression in breast cancers

Despite the presence of high levels of Notch receptors and ligands, we failed to detect Notch activity in normal breast tissue. We reasoned that this could be due to the presence of negative modulators of the pathway such as Numb. Numb inhibits Notch signaling by targeting Notch for ubiquitination followed by proteasome mediated degradation in conjunction with another protein, Itch [32]. Immunohistochemical analysis for Numb expression indeed revealed an abundant expression of Numb protein in all the normal tissues analyzed (Fig. 2B). Expression of Numb in breast cancers, however, seemed to be varied. We found the existence of Numb positive as well as Numb negative breast cancers (Fig. 2B and 2C). A subset of Numb positive tumors (21/36) were also positive for cleaved Notch1 and Hes1/5 (Additional file 3: Fig. 2), suggesting that Numb-resistant activation of Notch pathway may occur in breast carcinogenesis. This finding is in keeping with the results published by an earlier study [24], suggesting that negative regulation of Notch by Numb may be lost in breast cancers. We additionally found that DCIS also have heterogeneity with regard to Numb expression, with presence of both Numb positive and negative cases (data not shown). Thus, high levels of Numb in normal breast tissue may keep the Notch pathway in check, while in a large number of breast cancers, this negative regulation may be compromised.

### Notch signaling cooperates with the Ras/MAPK pathway to promote breast carcinogenesis

The expression of cleaved Notch and its downstream targets in early stages of breast cancer suggests that Notch functions may be involved in the transformation of initiated breast cells into cancerous cells. To gauge this, we utilized HMLE breast epithelial cells that have been generated by the introduction of Simian Virus 40 early region and the catalytic domain of human telomerase into human mammary epithelial cells [33]. We undertook retroviral-mediated gene delivery to introduce constructs over-expressing constitutively active Notch1 (AcN1) in HMLE cells (see methods).

**Figure 3:**
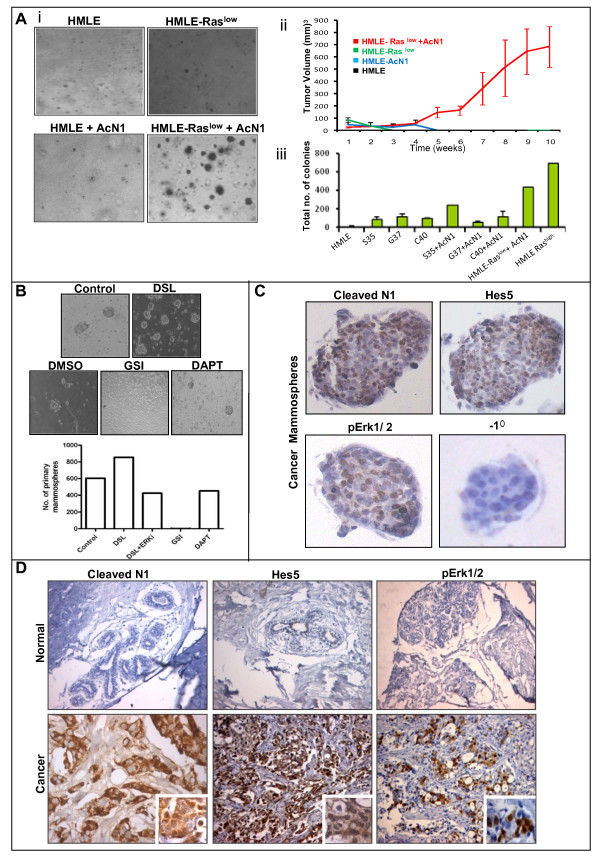
**Notch-Ras cooperation in breast carcinogenesis**: **(A) i**. Figure represents soft agar colony formation assay of HMLE, HMLE-AcN1, HMLE-Ras^low ^and HMLE-Ras^low^-AcN1 cells. **ii**. Graph shows tumor growth kinetics in nude mice for HMLE with (blue) & without (black) AcN1, and HMLE-Ras^low ^cells with (red) & without AcN1 (green). **iii**. Graph represents soft agar colony formation of HMLE cells expressing various Ras effector loop mutants alone, or in combination with AcN1. HMLE-Ras^high ^cells were used as positive control. **(B) **Photomicrographs and bar graphs represent the effect of Notch activation (using DSL peptide), Notch inhibition (using GSI and DAPT) and inhibition of MAPK (using MEK1 inhibitor) on mammosphere formation; magnification 10×. (**C**) Photomicrographs represent immunostaining of cancer mammospheres for cleaved Notch1, Hes5 and phosphoErk1/2; magnification 40×. **(D) **Photomicrographs represent staining of normal and cancerous breast tissue for cleaved Notch1, Hes5 and phosphoErk1/2; magnification 20×. Insets represent higher magnification (40×) revealing nuclear reactivity.

While HMLE cells expressing low levels of AcN1 (Additional file 5: Fig. 4) failed to form colonies in soft agar (Fig. 3A-i), or generate tumors when injected sub-cutaneously into nude mice (Fig. 3A-ii), cells expressing high levels of Notch1 could not be selected for as they exhibited massive cell death even prior to drug selection. It is possible, however, that HMLE cells may be able to tolerate high levels of AcN1 in the presence of other signaling pathways that may cooperate with Notch to mediate transformation. One likely candidate for cooperation with Notch signaling in the context of breast cancers is Ras. Even though Ras mutations are not prevalent in breast cancers, overexpression of its upstream tyrosine kinases, such as EGFR and Her2 neu, or constitutive activation of its downstream components leading to the activation of Ras signaling have been commonly observed in breast tumors [34,35]. Accordingly we assessed for Notch-Ras cooperation in HMLE cells.

It has been previously demonstrated that HMLE cells expressing low levels of oncogenic Ras (HMLE-Ras^low ^cells) failed to be transformed [33]. The HMLE-Ras^low ^cells, thus, provided a good system to investigate the Notch-Ras cooperation in breast cell transformation. Infection of HMLE-Ras^low ^cells with retroviruses expressing AcN1 enabled selection for high level expression of AcN1 (Additional file 5: Fig. 4), indicating that in the background of oncogenic Ras signaling, constitutively active Notch1 proteins are better tolerated in HMLE cells. When gauged for transformation, HMLE cells expressing both Ras^low ^and AcN1 generated efficient colonies in soft agar, while the parental HMLE-Ras^low ^cells failed to do so (Fig. 3A-i). Similarly, when injected subcutaneously into the flanks of nude mice, palpable tumor was observed starting at five weeks only when HMLE cells expressing both Ras and AcN1 were injected, while the control and parental cells failed to form tumors even after 10 weeks (Fig 3A-ii.). Thus, these findings clearly indicate that the Notch and Ras pathways functionally cooperate to transform immortalized breast epithelial cells.

Ras affects a number of cellular processes such as, proliferation, survival, and apoptosis, which are important in tumorigenesis. Upon activation, the effector loop domain of Ras interacts with downstream signaling components termed as Ras effectors. The three main Ras-effectors implicated in tumorigenesis are the PI3K, Raf, and Ral-GEFs [36]. In order to find out which of these effector pathways is involved in cooperating with the Notch pathway in the context of breast cell transformation, we made use of the Ras effector loop mutants that bind to, and specifically activate, only one of the pathway effectors. HMLE cells carrying the S35, C40 and G37 mutants of oncogenic Ras, that activate the MAPK, PI3K and Ral-GEFs respectively [35,37], were used for this study. Each of these cell lines was infected with AcN1-expressing virus simultaneously. Drug selection of HMLE-S35 and HMLE-C40 cells with puromycin resulted in the appearance of several colonies that grew rapidly. However, HMLE-G37 cells yielded very few colonies after drug selection, which grew slowly, suggesting that in the background of MAPK and PI3K pathways, Notch activation is better tolerated compared to those having Ral-GEF activation. When the Notch-expressing cells in all three mutant Ras backgrounds were subjected to soft agar colony formation analysis, we found that the HMLE-S35 cells yielded two and a half times more colonies compared to others (Fig. 3A-iii), indicating that the Notch pathway may collaborate with the Ras/MAPK pathway in mediating breast cancer pathogenesis. To further confirm the requirement of Ras signaling for Notch-mediated transformation of HMLE cells, we inhibited the MAPK pathway in HMLE-Ras^low ^cells expressing AcN1. Indeed, addition of UO126, a pharmacological inhibitor of the MAPK pathway, completely abrogated soft agar colony formation (data not shown). Thus, active signaling by both Ras and Notch is essential for transformation of breast cells.

We next investigated if the *in vitro *Notch-Ras cooperation has significance in the context of naturally arising tumors. Emerging evidence points to the role of stem cell maintenance in the context of carcinogenesis [38-40]. Notch signaling is involved in the regulation of stem cell self-renewal in several systems including the breast [9,39,41,42]. Both normal and cancerous breast stem cells can be enriched when cultured as mammospheres [43-45]. Indeed activation of the Notch pathway increased mammosphere forming potential, while its inactivation using gamma secretase inhibitor I (GSI I) completely abrogated sphere formation (Fig. 3B). A recent study, however, demonstrated that GSI may have cytotoxic effects on breast cells owing to proteasome inhibition [46]. In order to demonstrate the specific involvement of Notch signaling in mammosphere development, the Notch inhibitor, DAPT, was added. This resulted in reduction in the number of mammospheres, and not complete abrogation, as seen with GSI (Fig. 3B), thus, indicating the importance of Notch signaling in breast stem cell self renewal. In order to see if the Notch-Ras/MAPK cooperation is involved in stem cell maintenance in breast cancers, we analyzed the activation status of the two pathways using antibodies against cleaved Notch1 and phosphorylated forms of Erk1/2, the downstream targets of the Ras-MAPK pathway. In primary breast cancer-derived mammospheres, we detected intense nuclear staining for both cleaved Notch1 as well pErk1/2 (Fig. 3C). Furthermore, inhibition of the MAPK pathway using a specific MEK inhibitor (PD 98059) reduced mammosphere formation even in the presence of the Notch activator, suggesting that Notch-Ras cooperation may play a critical role in breast cancer-stem cell maintenance(Fig. 3B).

In order to further assess the *in vivo *relevance of the Notch-Ras/MAPK cooperation, we analyzed whether breast tumors that were positive for Notch activity (Fig. 2A) also showed MAPK activity. We found that a subset of cases showing high Notch activation, as detected by cleaved Notch and/or Hes5 staining, were also positive for nuclear phospho Erk 1 and 2 (13/24) (Fig. 3D). This association between Notch and Ras-MAPK expression was significant (p < 0.05) as analyzed by spearman's correlation test. This subset of breast cancers with both active notch and ras-mapk signaling were largely aggressive grade III carcinomas with high node positivity (indicative of increased risk of metastasis), further suggesting that the Notch-Ras cooperation in breast cancers may lead to poor prognosis.

## Discussion and Conclusion

The interaction of various signaling pathways in the development of breast cancer has been a subject of intense study for many years. In this study, we demonstrate that several Notch receptors and ligands are overexpressed in breast cancers, the Notch pathway is active in a large number of breast cancers, and it functionally interacts with the Ras/MAPK signaling pathway for mediating transformation.

### Expression of Notch receptors and ligands

Our results revealed a general increase in the expression of several Notch proteins (Notch 1,2 and 4) and ligands (Jagged1, 2, Dll1 and 4) in breast cancers compared to normal breast tissue, suggesting that the Notch pathway may contribute to breast cancer pathogenesis. While we failed to detect Notch3 and Delta-like1 in normal tissues, they were expressed at high to moderate levels in a subset of breast cancers. Interestingly, multiple Notch receptors and ligands were upregulated in a given cancer tissue (Additional file 3: Fig. 2). For eg., several breast cancers had high expression levels of both Notch1 and Notch3. Whether the output of different Notch receptor activation has different functions in tumorigenesis remains to be investigated.

Interestingly, studies using murine models of T-cell leukemia have shown that overexpression of the Notch3 intracellular domain antagonizes the tumorigenic effects of Notch1 [47]. In ErbB-2 negative breast cancers, downregulation of Notch3, but not Notch1, suppressed cell proliferation leading to apoptosis [48]. Thus, further dissection of the interplay between various receptors of the Notch pathway is required to better understand the process of breast cancer development.

Interestingly, we also detected the N-terminus of Notch3 in the nucleus of cancer cells. While this could represent either the full-length form of the protein or only the N-terminus, the process by which such nuclear accumulation occurs is unknown. As the N-terminus lacks a nuclear localization signal, it is tempting to speculate that it might be part of a bigger complex that is actively transported into the nucleus. Further experiments that address both the mechanism of transport and the significance of such nuclear accumulation are required to better understand their role in breast cancers.

### Aberrant activation of the Notch pathway is an early event

The timing of activation of the Notch pathway is very specific to the type of tumor involved. In cancers of the uterine cervix, Notch activation occurs during the transition from *in situ *carcinoma (CIN III) to frank invasive cancer [37,49]. On the other hand, chromosomal translocations that generate active Notch receptors initiate tumorigenesis in T-ALLs [13]. In the context of breast cancers, we detected evidence for the activation of the Notch pathway, as seen by the expression of both cleaved Notch and Hes1/5, starting as early as hyperplasia and DCIS, indicating that Notch activation may be an initial trigger in the onset of breast cancers.

We observe strong nuclear positivity of cleaved Notch1 in several breast cancer tissues. This is frequently accompanied by the expression of downstream transcriptional targets such as Hes1/5. In addition, we also detect the presence of large amounts of cytosolic cleaved Notch1. This is in keeping with the observation in several other tissues using the same antibody [25,50]. This could imply that cleaved Notch1, in addition to its trans-activation functions in the nuclei, may have additional functions in the cytoplasm. Such functions of Notch have indeed been reported elsewhere. The activation of PKB/Akt by cleaved Notch1 [51], and the interaction of cytosolic Notch with PI3K and p56lck in the cytoplasm has been previously reported [52]. Furthermore, CBF1-independent, deltex-dependent cytosolic functions of Notch have also been observed [7]. It will be interesting to dissect the nuclear and cytosolic functions of Notch1 in the context of breast tumorigenesis.

### Cooperation of Notch and Ras/MAPK pathway

Interactions between the Notch and Ras pathways have been reported to have both antagonistic and synergistic effects in different contexts [53]. Previous studies have demonstrated a correlation between the expression of Ras and Notch1 in breast cancers [54] suggesting a possible interaction between these two pathways. We demonstrate a functional cooperation between constitutively active Notch1 and Ras in the transformation of immortalized breast epithelial cells as well as in breast stem cell self-renewal. We also detect evidence of both Ras and Notch pathway activation in the context of naturally arising breast cancers, and such tumors presented with high node positivity, indicating that co-activation of these two pathways may serve as a prognostic marker for breast cancer. An epistasis analysis of their interaction in the context of tumorigenesis would provide valuable insight into their individual and collective functions.

Taken together, our experiments place Notch as a key player in breast carcinogenesis. Therapeutic interventions at various levels, such as ligand-receptor interaction, Notch nuclear translocation, and Notch-Ras cooperation, stand to be exploited in treating breast cancers.

## Methods

### Immunohistochemistry and tissue samples

Human breast tissue sections were obtained from tumour blocks archived in the Department of Pathology at the Kidwai Memorial Institute of Oncology (KMIO). Briefly, the paraffin embedded tissue sections were deparaffinized in xylene and successively rehydrated. Peroxidase activity was quenched using 5% hydrogen peroxide. The antigen retrieval was done by exposing the sections to steam at high pressure in a conventional pressure cooker by placing the sections in 10 mM freshly prepared sodium citrate buffer, pH 6.0. Alternatively, sections were boiled in 10 mM sodium citrate buffer for 20 minutes in a water bath. After blocking the nonspecific binding with 4% non fat dry milk, the sections were incubated overnight with the respective primary antibodies at 4°C: Notch1, Notch2, Notch3, Notch4, Delta-like1, Delta-like4, Jagged1, Jagged2, Hes5, Hes1, Numb, cleaved Notch1 antibodies (Santa Cruz Biotechnology, Inc, CA.) and pErk1&2 (Cell Signaling Technology, Inc, CA). The secondary anti-mouse and anti goat antibodies and the ABC color development kit was procured from Bangalore Genei, India.

#### Evaluation of immunohistochemistry

Immunoreactivity was considered significant when the characteristic immunostaining was observed in more than 10% of the cells. The intensity of staining was graded from 1 to 4, with the lowest staining marked as 1+ to the highest staining marked as 4+ (Additional file 2: Fig. 1). Batch to batch variation in staining intensity was compensated by including each time a positive control slide that stained intensely for Jagged1. This tissue was assigned an intensity of 4+, and the rest of the samples were graded with respect to this control. Sections were evaluated by the pathologist (RVK) at KMIO.

### Plasmid constructs

The Ras retroviral expression constructs [33] and individual Ras effector loop mutants [37] have been previously described. The BamH1 and Sal1 fragment of the intracellular domain of Notch 1 from pBABEpuro-hN1C (gift from A. Capobianco) was ligated into pBABE-puro and pBabe hygro retroviral expression constructs and over-expression confirmed by Western blot analysis. As shown previously for Ras [33], the pBABE puro construct yielded high level expression of AcN1, while the pBABE-hygro construct yielded low level expression **(Additional file **5: **Fig. 4**).

### Cell culture and transfection conditions

The HMLE cells were generated by the exogenous introduction of SV40 ER and hTERT into HMECs [33]. HMLE cells were grown in DME-F12 media supplemented with insulin, epidermal growth factor (EGF), and hydrocortisone under standard tissue culture conditions. Retroviral infections of HMLE were performed (as described earlier) for over expressing constitutively active Notch1 and drug selection was used to purify polyclonal-infected populations [37].

### Anchorage-independent growth assay

Soft agar assays were performed as described earlier [37]. Individual cell lines were seeded in triplicates at three different dilutions ranging between 1 × 10^4 ^to 5 × 10^5^. Each experiment was repeated 2-3 times. Colonies were photographed between 18-24 days at a final magnification of 20× under phase contrast microscope.

### Mammosphere assay

Primary breast tissue was obtained from KMIO in keeping with the ethical guidelines set up by both the institutions, and with informed patient consent. Mammospheres were generated as described in Dey et al., [43]. For Notch activation, 2.5 × 10^5 ^organoid-derived single cells were seeded for mammosphere formation and incubated with 100 nM water-soluble DSL peptide (CDDYYYGFGCNKFCRPR; Genscript Corp. USA) [42] for one week after which the primary mammospheres were counted. Notch inhibition was carried out for one week in the presence of 10 μM Γ- Secretase Inhibitor I (GSI I; Calbiochem) or 10 μM DAPT (Calbiochem). DMSO was used as a vehicle control for GSI. For inhibition of the MAPK pathway, Mek1 inhibitor (PD98059; Cell Signaling Technology) was added at 25 μM concentration.

### Tumorigenicity assay

Nude mice that were 6 to 8 week old were injected subcutaneously with 2 × 10^6 ^cells admixed with 50% Matrigel (Becton Dickinson, Palo Alto, California). Error bars represent mean tumor volume +/- SEM.

## Competing interests

The authors declare that they have no competing interests.

## Authors' contributions

SM carried out all IHC studies and participated in manuscript preparation, DS helped in study design, drafting, and critical revision of manuscript, RVK helped with IHC data analysis, DD carried out mammosphere assays, AR carried out soft agar and tumorigenecity experiments and drafted the manuscript. All the authors have read and approved the final manuscript.

## Appendix 1

### Methods

#### RT-PCR analysis

Total RNA was extracted from cultured cells using Tri-reagent (Sigma) and subjected to DNase I (Sigma) digestion to eliminate contaminating genomic DNA. RT reaction for 1 μg total RNA was performed using M-MuLV Reverse Transcriptase and 0.5 ug oligo(dT)_18_primer. PCR was carried out under standard conditions using following pairs of gene-specific primers:

Notch1:CAACATCCAGGACAACATGG, TTGTTAGCCCCGTTCTTCAG

Notch2:TTATGCAGGACCCGTTGTG, ACACTTTGCCCCATTCAGAC

Notch3:GGGAGTCCCTCAAGGCTATC, GATGGAGAGGAGGAGGGAAG

Notch4:GATAAATGGGGGAAAACTGC, GATCCCCAGTGGTTACGTTGG

Jagged1:ACAACACCACCAACAACGTG, GGGCACTTTCCAAGTCTCTG

Jagged2:AGGTGGAGACGGTTGTTACG, TTGCACTGGTAGAGCACGTC

Dll1:GCCTCAAGCCCACTGTCTAC, ACACACACACACACGCACAC

Dll3:CAATGGAGGCAGCTGTAGTG, TCAAAGGACCTGGGTGTCTC

Dll 4:CTATGGCCTGCATTGTGAAC, ACAGTAGGTGCCCGTGAATC

## Supplementary Material

Additional File 1 **Table 1**. RT-PCR analysis of Notch receptors and ligands in immortalized (MCF-10A, HBL100, and HMLE) and cancerous (MCF7, MDA MB 435, MDA MB 453, MDA MB 468, SW 613, T47D, HMLER) breast cell lines. Pink and blue depict the absence (-) and presence (+) of the transcripts, respectively. Click here for file

Additional file 2 **Fig. 1**. Quantification of immunohistochemical analysis. Photomicrographs show immunohistochemical staining of breast tissue sections representing different intensities graded between 1+ and 4+ based on visual observation. This gradation was used to evaluate intensities for all antigens. Negative control (-1°) represents staining in the absence of primary antibody; magnification 20×. Click here for file

Additional File 3 **Fig. 2**. The heat map shows cumulative and comparative staining of normal and cancer tissues for different antibodies and lymph node status. ND: Not Determined; Neg: Negative; NA: Not Available. Since case number 22 floated away, no staining could be performed on this. Click here for file

Additional file 4 **Fig. 3**. Photomicrographs represent immunostaining of adjacent areas of normal (N) and cancer (C) within the same section using antibodies against cleaved Notch1 and Hes5. Click here for file

Additional file 5 **Fig. 4**. Immunoblot analysis reveals expression of constitutively active, cleaved Notch1 expressed by pBABE-Hygro-AcN1 construct in HMLE cells, and pBABE-puro-AcN1 construct in HMLE-Ras^low ^cells. β actin was used as loading control. Click here for file
