# Supplementary material for: Cooperation of Notch and Ras/MAPK signaling pathways in human breast carcinogenesis
Source: Mol Cancer. 2009 Dec 23;8:128. doi: 10.1186/1476-4598-8-128 (PMC2809056; doi:10.1186/1476-4598-8-128)
Supplement: Additional File 1 — Table 1. RT-PCR analysis of Notch receptors and ligands in immortalized (MCF-10A, HBL100, and HMLE) and cancerous (MCF7, MDA MB 435, MDA MB 453, MDA MB 468, SW 613, T47D, HMLER) breast cell lines. Pink and blue depict the absence (-) and presence (+) of the transcripts, respectively. [file 1476-4598-8-128-S1.PPT]

## Slide 1
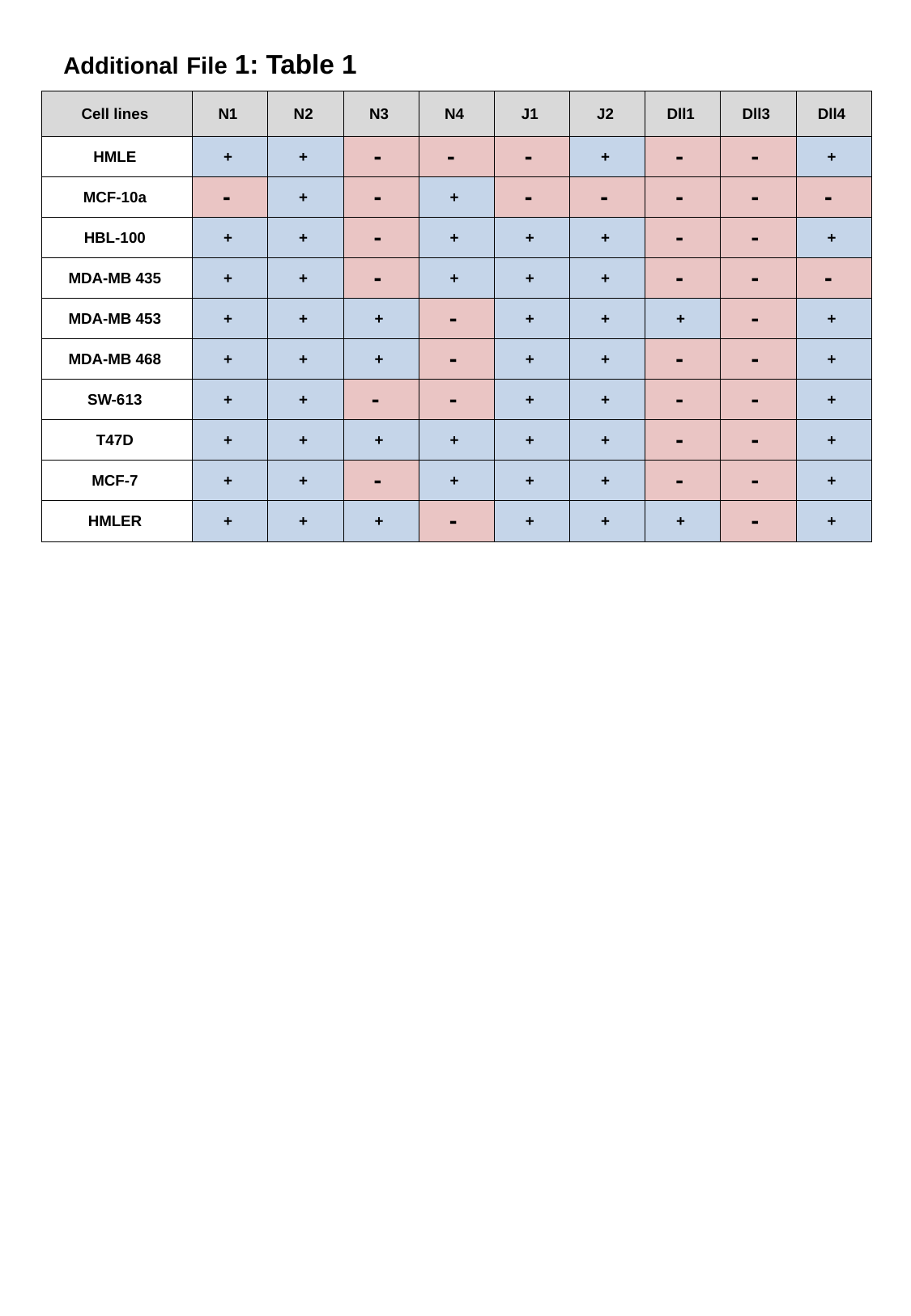

Additional File 1: Table 1
| Cell lines | N1 | N2 | N3 | N4 | J1 | J2 | Dll1 | Dll3 | Dll4 |
| --- | --- | --- | --- | --- | --- | --- | --- | --- | --- |
| HMLE | + | + | - | - | - | + | - | - | + |
| MCF-10a | - | + | - | + | - | - | - | - | - |
| HBL-100 | + | + | - | + | + | + | - | - | + |
| MDA-MB 435 | + | + | - | + | + | + | - | - | - |
| MDA-MB 453 | + | + | + | - | + | + | + | - | + |
| MDA-MB 468 | + | + | + | - | + | + | - | - | + |
| SW-613 | + | + | - | - | + | + | - | - | + |
| T47D | + | + | + | + | + | + | - | - | + |
| MCF-7 | + | + | - | + | + | + | - | - | + |
| HMLER | + | + | + | - | + | + | + | - | + |
