# Supplementary figures and images for: Cooperation of Notch and Ras/MAPK signaling pathways in human breast carcinogenesis
Source: Mol Cancer. 2009 Dec 23;8:128. doi: 10.1186/1476-4598-8-128 (PMC2809056; doi:10.1186/1476-4598-8-128)

## Slide 1
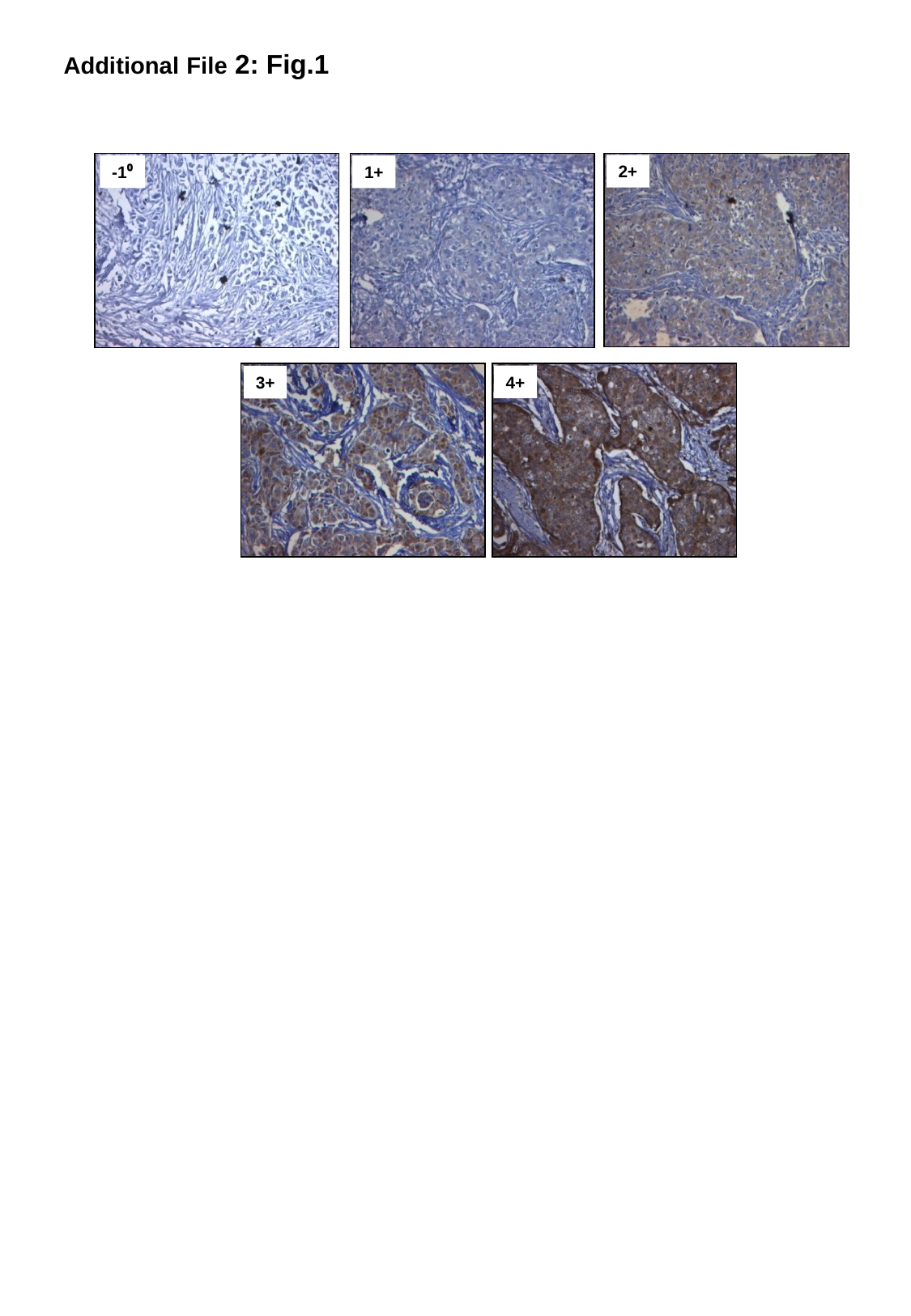

Additional File 2: Fig.1
2+
-1⁰
1+
4+
3+

Supplement: Additional file 2 — Fig. 1. Quantification of immunohistochemical analysis. Photomicrographs show immunohistochemical staining of breast tissue sections representing different intensities graded between 1+ and 4+ based on visual observation. This gradation was used to evaluate intensities for all antigens. Negative control (-1°) represents staining in the absence of primary antibody; magnification 20×. [file 1476-4598-8-128-S2.PPT]

## Slide 1
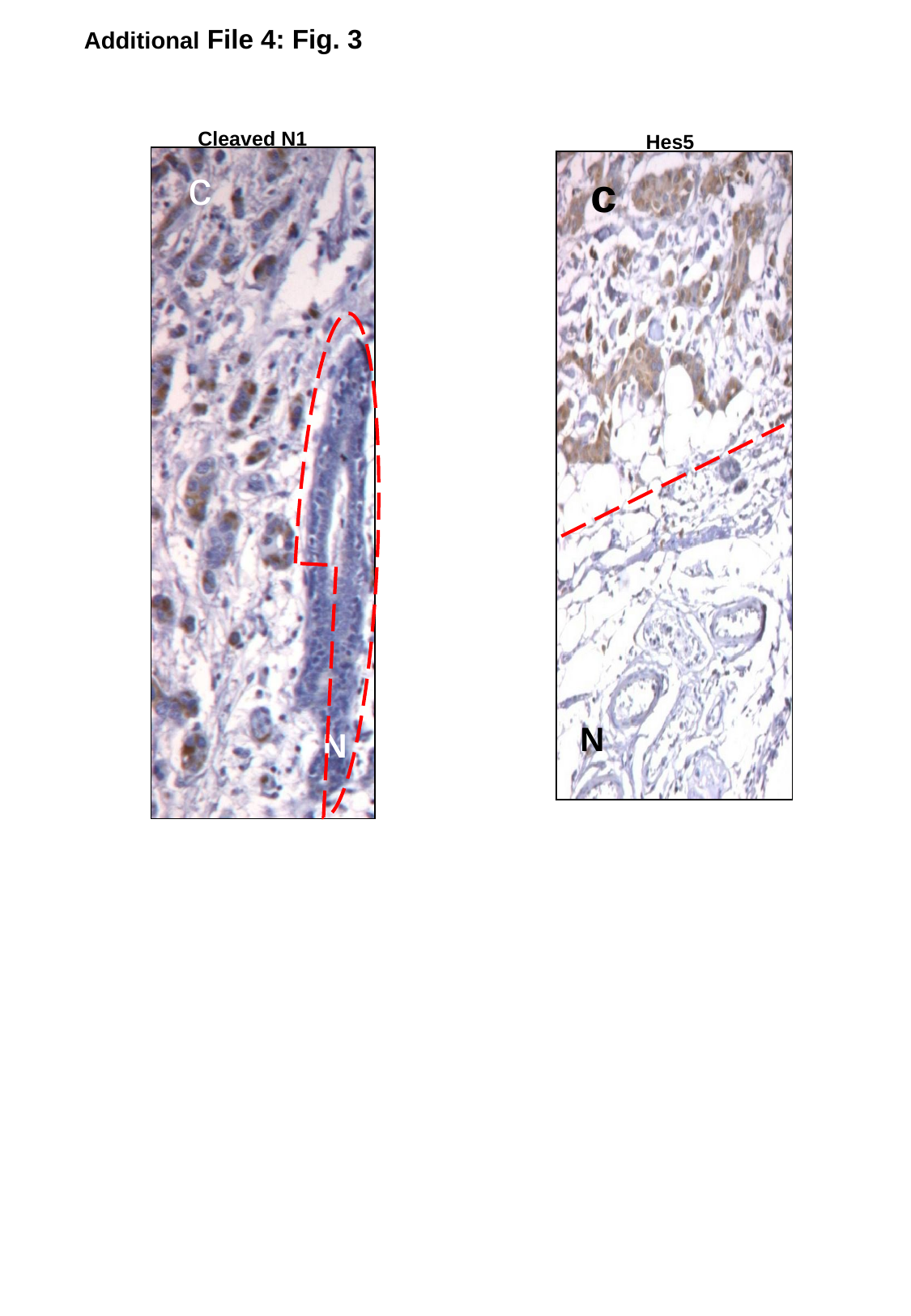

Additional File 4: Fig. 3
Cleaved N1
Hes5
c
c
N
N

Supplement: Additional file 4 — Fig. 3. Photomicrographs represent immunostaining of adjacent areas of normal (N) and cancer (C) within the same section using antibodies against cleaved Notch1 and Hes5. [file 1476-4598-8-128-S4.PPT]

## Slide 1
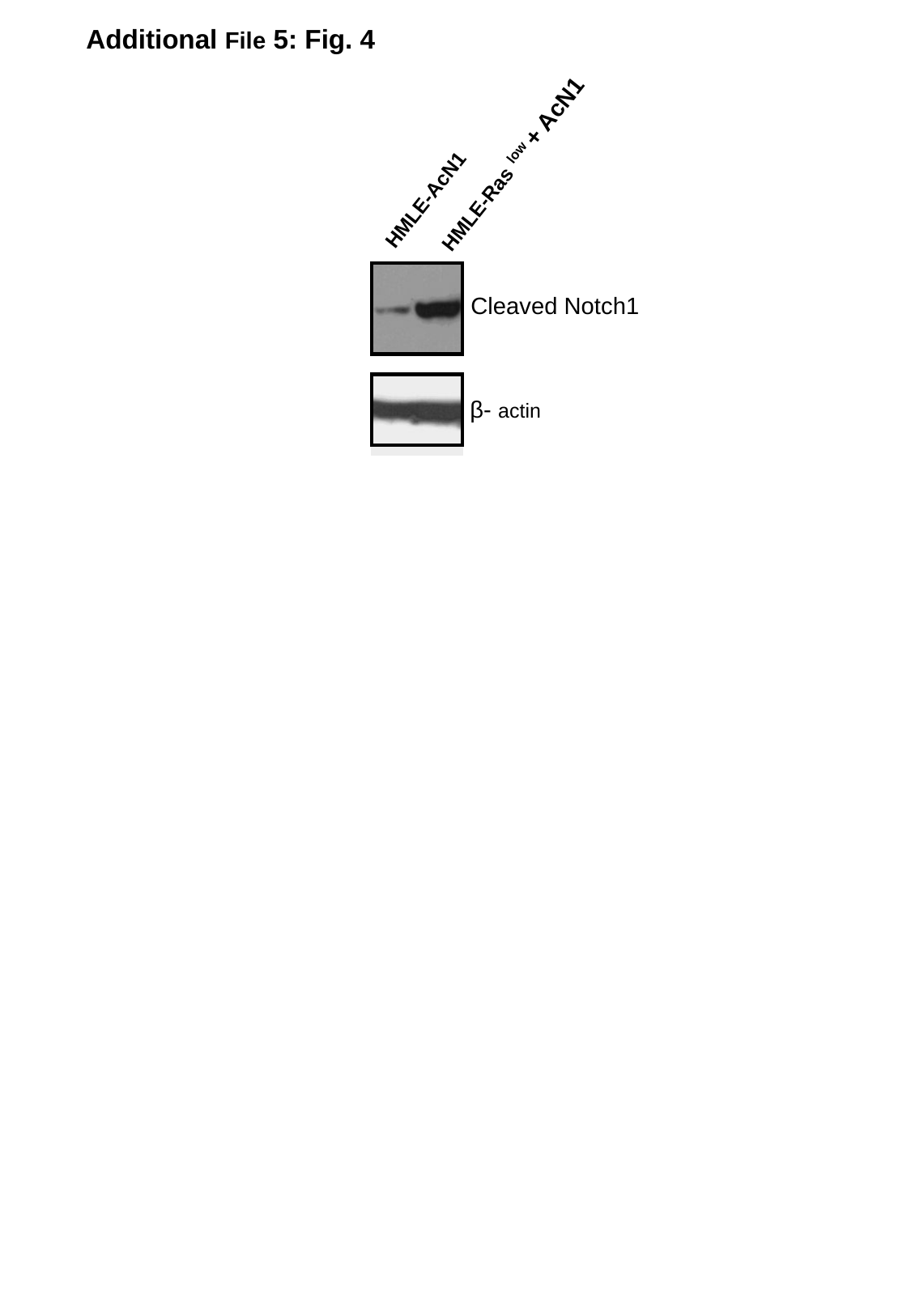

Additional File 5: Fig. 4
HMLE-Ras low + AcN1
HMLE-AcN1
Cleaved Notch1
β- actin

Supplement: Additional file 5 — Fig. 4. Immunoblot analysis reveals expression of constitutively active, cleaved Notch1 expressed by pBABE-Hygro-AcN1 construct in HMLE cells, and pBABE-puro-AcN1 construct in HMLE-Raslow cells. β actin was used as loading control. [file 1476-4598-8-128-S5.PPT]
