# Supplementary material for: Cooperation of Notch and Ras/MAPK signaling pathways in human breast carcinogenesis
Source: Mol Cancer. 2009 Dec 23;8:128. doi: 10.1186/1476-4598-8-128 (PMC2809056; doi:10.1186/1476-4598-8-128)
Supplement: Additional File 3 — Fig. 2. The heat map shows cumulative and comparative staining of normal and cancer tissues for different antibodies and lymph node status. ND: Not Determined; Neg: Negative; NA: Not Available. Since case number 22 floated away, no staining could be performed on this. [file 1476-4598-8-128-S3.PPT]

## Slide 1
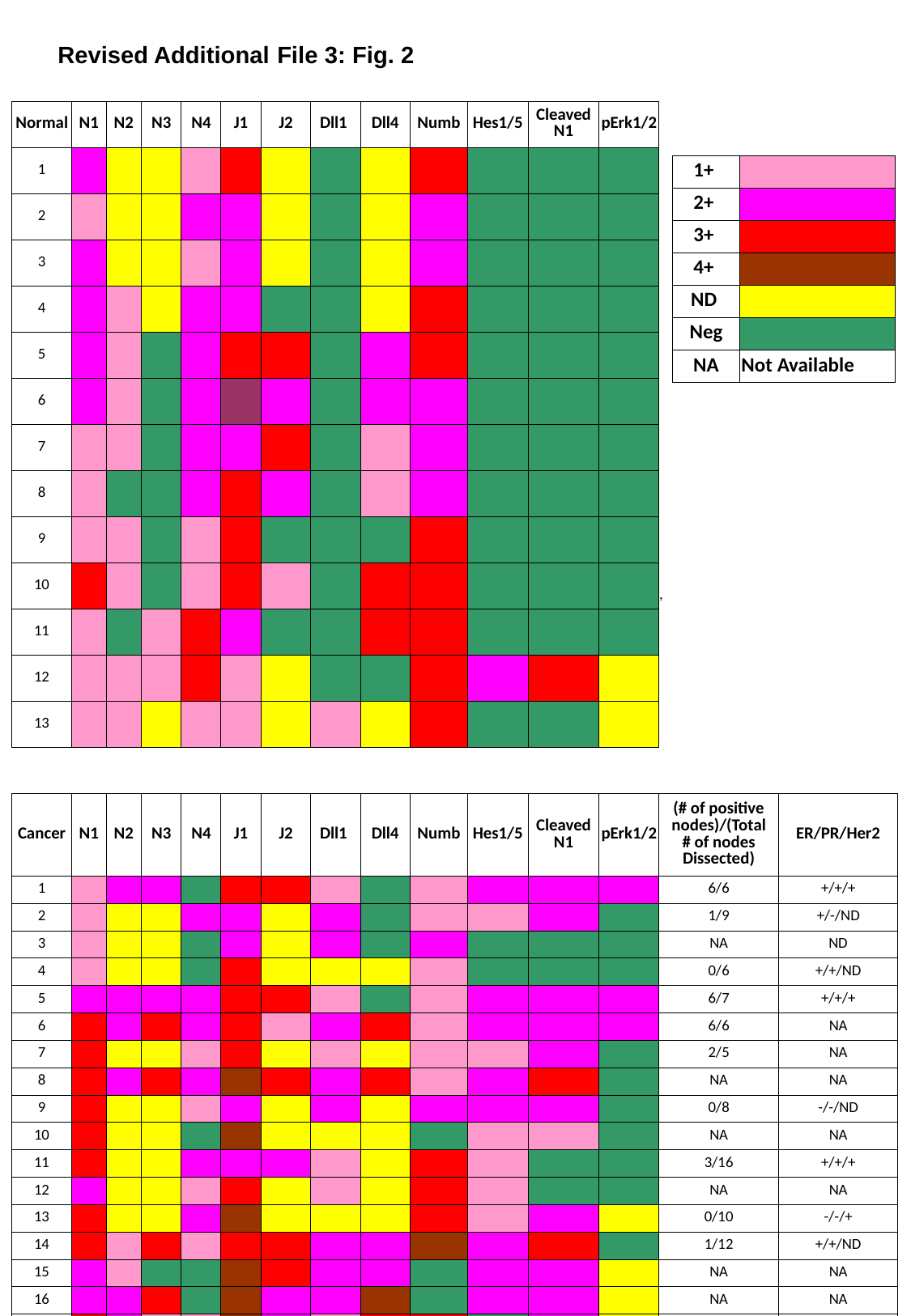

Revised Additional File 3: Fig. 2
| Normal | N1 | N2 | N3 | N4 | J1 | J2 | Dll1 | Dll4 | Numb | Hes1/5 | Cleaved N1 | pErk1/2 | | |
| --- | --- | --- | --- | --- | --- | --- | --- | --- | --- | --- | --- | --- | --- | --- |
| 1 | | | | | | | | | | | | | | |
| 2 | | | | | | | | | | | | | | |
| 3 | | | | | | | | | | | | | | |
| 4 | | | | | | | | | | | | | | |
| 5 | | | | | | | | | | | | | | |
| 6 | | | | | | | | | | | | | | |
| 7 | | | | | | | | | | | | | | |
| 8 | | | | | | | | | | | | | | |
| 9 | | | | | | | | | | | | | | |
| 10 | | | | | | | | | | | | | , | |
| 11 | | | | | | | | | | | | | | |
| 12 | | | | | | | | | | | | | | |
| 13 | | | | | | | | | | | | | | |
| | | | | | | | | | | | | | | |
| Cancer | N1 | N2 | N3 | N4 | J1 | J2 | Dll1 | Dll4 | Numb | Hes1/5 | Cleaved N1 | pErk1/2 | (# of positive nodes)/(Total # of nodes Dissected) | ER/PR/Her2 |
| 1 | | | | | | | | | | | | | 6/6 | +/+/+ |
| 2 | | | | | | | | | | | | | 1/9 | +/-/ND |
| 3 | | | | | | | | | | | | | NA | ND |
| 4 | | | | | | | | | | | | | 0/6 | +/+/ND |
| 5 | | | | | | | | | | | | | 6/7 | +/+/+ |
| 6 | | | | | | | | | | | | | 6/6 | NA |
| 7 | | | | | | | | | | | | | 2/5 | NA |
| 8 | | | | | | | | | | | | | NA | NA |
| 9 | | | | | | | | | | | | | 0/8 | -/-/ND |
| 10 | | | | | | | | | | | | | NA | NA |
| 11 | | | | | | | | | | | | | 3/16 | +/+/+ |
| 12 | | | | | | | | | | | | | NA | NA |
| 13 | | | | | | | | | | | | | 0/10 | -/-/+ |
| 14 | | | | | | | | | | | | | 1/12 | +/+/ND |
| 15 | | | | | | | | | | | | | NA | NA |
| 16 | | | | | | | | | | | | | NA | NA |
| 17 | | | | | | | | | | | | | NA | NA |
| 18 | | | | | | | | | | | | | NA | NA |
| 19 | | | | | | | | | | | | | 6/10 | +/+/+ |
| 20 | | | | | | | | | | | | | NA | NA |
| 21 | | | | | | | | | | | | | 6/9 | +/+/ND |
| 22 | | | | | | | | | | | | | | |
| 23 | | | | | | | | | | | | | 0/6 | NA |
| 24 | | | | | | | | | | | | | 12/15 | +/-/+ |
| 25 | | | | | | | | | | | | | 6/9 | NA |
| 26 | | | | | | | | | | | | | 6/11 | -/+/ND |
| 27 | | | | | | | | | | | | | 0/8 | NA |
| 28 | | | | | | | | | | | | | 16/16 | NA |
| 29 | | | | | | | | | | | | | 0/7 | NA |
| 30 | | | | | | | | | | | | | NA | NA |
| 31 | | | | | | | | | | | | | 11/13 | NA |
| 32 | | | | | | | | | | | | | 5/7 | NA |
| 33 | | | | | | | | | | | | | NA | NA |
| 34 | | | | | | | | | | | | | 9/10 | +/+/ND |
| 35 | | | | | | | | | | | | | 3/8 | -/-/ND |
| 36 | | | | | | | | | | | | | NA | NA |
| 37 | | | | | | | | | | | | | NA | NA |
| 1+ | |
| --- | --- |
| 2+ | |
| 3+ | |
| 4+ | |
| ND | |
| Neg | |
| NA | Not Available |
